# COVID-19 as a Potential Cause of Muscle Injuries in Professional Italian Serie A Soccer Players: A Retrospective Observational Study

**DOI:** 10.3390/ijerph191711117

**Published:** 2022-09-05

**Authors:** Giuseppe Annino, Vincenzo Manzi, Anas Radi Alashram, Cristian Romagnoli, Mattia Coniglio, Niloofar Lamouchideli, Marco Alfonso Perrone, Dolores Limongi, Elvira Padua

**Affiliations:** 1 Department of Systems Medicine, Faculty of Medicine and Surgery, University of Rome “Tor Vergata”, 00133 Rome, Italy; 2 Centre of SpaceBio-Medicine, University of Rome “Tor Vergata”, 00133 Rome, Italy; 3 Department of Humanities Science, Pegaso Open University, 80143 Naples, Italy; 4 Hellas Verona FC, Via Olanda 11, 37135 Verona, Italy; 5 Department of Physiotherapy, Middle East University, Amman 11831, Jordan; 6 Department for Life Quality Studies Rimini, Alma Mater University, 47521 Bologna, Italy; 7 Faculty of Medicine and Surgery, University of Rome “Tor Vergata”, 00133 Rome, Italy; 8 Department of Human Neuroscience, University of Rome “La Sapienza”, 00185 Rome, Italy; 9 University Sports Centre, University of Rome “Tor Vergata”, 00133 Rome, Italy; 10 IRCCS San Raffaele Pisana, 00166 Rome, Italy; 11 Department of Human Sciences and Promotion of the Quality of Life, San Raffaele Roma Open University, 00166 Rome, Italy

**Keywords:** COVID-19, soccer players, Italian Serie A, muscular injuries

## Abstract

The COVID-19 pandemic has shocked the entire planet. The soccer world has also suffered major upheavals, and many professional soccer players have been infected with the virus. The aim of this study was to evaluate the incidence of injuries in Italian Serie A professional soccer players before and during the COVID-19 pandemic. Methods: We evaluated the incidence of muscle injuries between four competitive seasons of the Italian Serie A (2016–2017, 2017–2018, and 2018–2019 pre-COVID-19 vs. 2020/2021 post-COVID-19) in professional soccer players. Results: Significant differences were found in muscular injuries between the post-COVID-19 season and the previous seasons (*p* < 0.001). The median split of the players’ positivity duration was of 15 days. The players’ long positivity (PLP) group showed a significant number of muscular injuries compared to the players’ short positivity (PSP) group (*p* < 0.0014, ES = 0.81, Large). The total teams’ days of positivity were significantly related to the total team number of muscular injuries (r = 0.86; CI 95% 0.66 to 0.94; *p* < 0.0001). In conclusion, this data showed that the competitive season post-COVID-19 lockdown has a higher incidence of muscle injuries in Italian Serie A soccer players compared to the pre-pandemic competitive season.

## 1. Introduction

The soccer sport demands intermittent actions that are performed at an intensity that the player’s muscular strength, power, and endurance represent important factors for high-level performance in this discipline [1,2,3]. Several neuromuscular and neurosensory circuitries are brought into play to develop elevated strength to respond adequately to these demands [4].

Considering that soccer players sometimes perform training sessions or matches not in optimal muscular conditions, the risk of injury could increase significantly in terms of quantity and frequency [5,6]. Some studies have observed a negative effect of fixture congestion on variables such as low- and moderate-intensity distance covered, perhaps suggesting that players employ pacing strategies to maintain high-intensity actions while underestimating the possible repercussions on injury prevention [7]. In this regard, the injury rate over the years has resulted in an increased absence from playing official matches with a consequent economic deficit and decreased physical performance [8,9].

Coronavirus disease 2019 (COVID-19) is a viral infection that develops following the access of severe acute respiratory syndrome-coronavirus-2 (SARS-CoV-2) to the respiratory tract [10]. SARS-CoV-2 sometimes induces severe inflammatory and oxidative stress, which injure pulmonary alveoli, resulting in the development of severe acute respiratory distress syndrome (ARDS), bilateral viral pneumonia, and respiratory failure [11]. Moreover, SARS-CoV-2 particles have been isolated from different body tissues, including the intestine, the central nervous system, the cardiac muscle, and the skeletal muscle [12]. Muscle pain is one of the key symptoms that develop during the first three days of infection in people who become hospitalized due to contracting SARS-CoV-2 [13,14,15].

The exact mechanism of muscle damage in COVID-19 patients, as well as the long-term consequences of muscle injury in disease survivors, are unclear. However, it has been suggested that muscle loss in COVID-19 patients is an outcome of a wide range of interrelated factors, among which the excessive production of proinflammatory cytokines in hypercatabolic conditions, associated with oxidative stress, which promotes the production of corrosive molecules causing severe myocyte damage, is certainly the most important. COVID-19 has affected the entire world population, including elite athletes, with different prognoses, from mild to severe disease up to death.

In this context, many athletes were affected by COVID-19, prevalently in a mild form [16]. Among the variety of symptoms reported by the general population, muscle soreness, fatigue, and weakness were very frequent, making it highly likely that the COVID-19 disease could induce muscle tissue damage and more severe clinical manifestations in the lung, cardiac, and neural tissues [17]. Thus, it is worth noting that a recent study reported an increase in soft tissue injuries among England soccer premier league players during the COVID-19-impacted championship of 2020–2021 in comparison to the three preceding seasons [3,18], although the rate of injuries in the infected players was not analyzed.

This study aimed to assess whether the injury rates had increased after the last lockdown period by comparing the Italian A series of soccer after the pandemic lockdown against the three pre-lockdown Serie A seasons and whether the frequency of muscular injuries was greater in players infected by the SARS-CoV-2.

## 2. Materials and Methods

Similar to other studies [5,18,19], we used the transfer market site (https://www.transfermarkt.com/; data collection started in October 2021). Transfermarkt is a German website containing football information, rankings of numerous leagues from around the world, match results, transfers player careers, company data, injury data, information on sports agents, as well as a section dedicated to transfer market news [20]. The information contained in the site comes from the clubs, as the databases are formed with the collaboration of some members of the various clubs. In this context, the positive days were also communicated by the clubs by law except for the level of symptoms of the players affected by COVID-19 and for those not analyzed in this study.

In this media-based study, all the injuries recorded in the last four Italian Serie A championships (S.S.2016–2017, 2017–2018, 2018–2019 pre-COVID-19, and 2020–2021 SARS-CoV-2 post-pandemic lockdown seasons), performed with the same conducting criteria, were analyzed. The differences in the conducting of the 2019–2020 championship due to pandemic viral diffusion and consequent lockdown restrictions (three months) were excluded from the present analysis. Injuries were considered for those that fell within this definition: “damage that occurs in training or a match that prevents the player from participating in the next training session or match” [8,21,22]. Three authors independently examined the online TransferMarkt.us database, one of which analyzed the results provided, assessing the inter-raters’ agreement. The number of injuries and the injury characteristics were analyzed and divided into muscle and joint injuries, and, excluding all injuries with a certain external cause (e.g., contact), those with non-contact injuries were selected for statistical analysis. In cases of disagreement, the decision was made by a senior author experienced in sports medicine. Therefore, to be registered, an injured player did not have to take part in at least one official match. In addition, we considered all muscular and articular injuries in the Italian A series soccer. Subsequently, in the 2020–2021 championship, we distinguished the player affected by COVID-19 and their relative post-COVID-19 injuries after the positivity period.

### Statistical Analyses

Data are presented as mean ± SD and confidence intervals at a 95% level (95% CI). Before any parametric statistic was performed, the assumption of normality was tested with the Shapiro–Wilk test on each variable. The One-Way ANOVA analysis of variance was used to identify the differences in the total number of players examined in the total number of official games played, as well as in the muscular and articular injuries between the soccer seasons of 2016–2017, 2017–2018, 2018–2019 (pre-COVID-19), and soccer season 2020–2021 (SARS-CoV-2 post-pandemic lockdown seasons). Bonferroni’s post hoc test of critical difference was used to locate significance between means. The soccer players (season 2020–2021) were divided into 2 groups according to the median days of positivity (players’ long positivity PLP and players’ short positivity PSP) using the ‘median split’ technique [8,23]. The players obtaining the median days of positivity value were excluded. Two groups with the same number of participants (n = 35) were obtained. Then, an unpaired t-test was utilized to determine any significant difference in players’ number of muscular injuries between PLP and PSP. The magnitude of the mean difference was shown as effect size (ES) and was interpreted according to the criteria used by Cohen [24]. Effect sizes of >0.8, between 0.8 and 0.5, between 0.5 and 0.2, and <0.2 were considered as large, moderate, small, and trivial, respectively. The association between total teams’ days of positivity and total teams’ number of muscle injuries was evaluated using Pearson’s correlation coefficient. Magnitude of correlation was qualitatively ranked according to Hopkins [25] as follows: trivial r, 0.1, small 0.1, r, 0.3, moderate 0.3, r, 0.5, large 0.5, r, 0.7, very large 0.7, r, 0.9, nearly perfect r, 0.9, and perfect r = 1. Significance was set at *p* < 0.05, and all analyses were performed using SPSS 25 (SPSS Inc., Chicago, IL, USA).

## 3. Results

The total number of players examined and total official games played between the soccer seasons 2016–2017, 2017–2018, 2018–2019 (pre-COVID-19), and soccer season 2020–2021 (SARS-CoV-2 post-pandemic lockdown seasons) are presented in Table 1.

**Figure 1:**
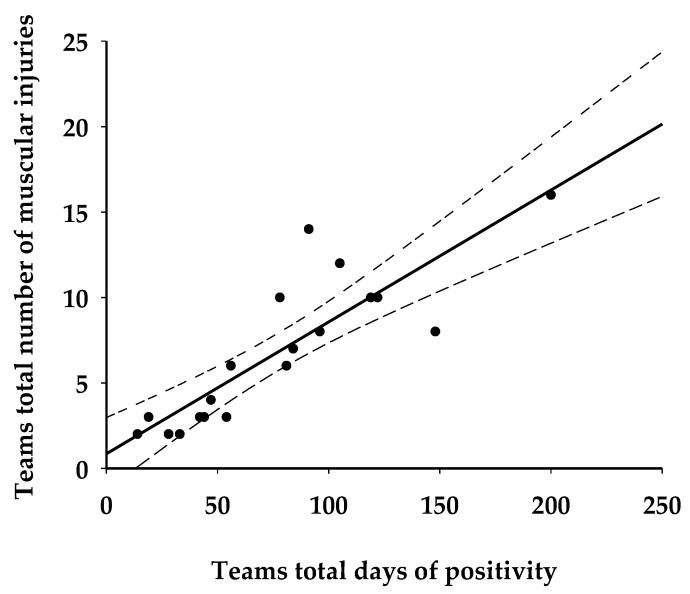
Relationship between teams’ total days of positivity and teams’ total number of injuries (r = 0.86; CI 95% 0.66 to 0.94; *p* < 0.0001).

Moreover, an analysis of the injury that occurred between the soccer season 2016–2017, 2017–2018, 2018–2019 (pre-COVID 19), and soccer season 2020–2021 (COVID-19 post-pandemic lockdown seasons) are presented in Table 1 and Table 2. Significant season differences were found for muscular injury (*p* < 0.001) but not for articular injury (*p* > 0.05). Overall, 318, 289, 386, and 574 muscular injuries were observed across the 2016/2017, 2017/2018, 2018/2019, and 2020/2021 seasons, respectively. The median split of the players’ positivity duration was of 15 days. The players’ long positivity group showed a significant number of muscular injury respect to players’ short positivity (*p* < 0.0014, ES = 0.81, Table 3). Interestingly, the total teams’ days of positivity was significantly related to the total team number of muscular injury (r = 0.86; CI 95% 116 0.66 to 0.94; *p* < 0.0001, Figure 1).

## 4. Discussion

This study showed that all of the pre- and post-pandemic lockdown championships analyzed were performed regularly and in the same temporal arch, showing no significant differences in terms of players per team and number of played matches (Table 1).

In addition, the results of the data analysis suggest that the incidence of muscle injuries increased in the Serie A soccer season 2020–2021 (SARS-CoV-2 post-pandemic lockdown season), and that the teams’ total days of positivity is significantly associated with the teams’ total number of injuries. In fact, significant differences in muscle injury were found between the Serie A seasons preceding the pandemic lockdown and the 2020–2021 season characterized by the circulation of the virus (Table 2 and Table 3). Moreover, as shown in Table 4, players affected by long positivity seem to be more inclined to incur a non-contact muscular injury than players affected by short positivity.

No changes were observed in particular injuries. These results seem to be in line with what has been observed in the English premier league [3,18]. However, we extended these findings by showing that the incidence in the number of muscle injuries correlates to the level of impairment induced by the viral infection, as assessed by the time duration needed for the COVID-19 negativization experienced by athletes before their return to play (RTP). Players who underwent a longer time before RTP seem to be more exposed to muscle injury and, in some cases, to repeat injuries. Interestingly, the time before RTP, expressed by the overall days of positivity of the infected players belonging to all teams, showed a positive correlation with the number of injuries, as illustrated in Figure 1. To date, the potentially harmful morphological and functional alterations of the SARS-CoV-2 virus on muscle tissue have been scantly investigated, even though muscle aches and fatigue are frequently reported in association with other symptoms [18,26].

In this context, it is important to note that the ACE2 receptor is widely expressed in the human body, and the musculoskeletal system is not an exception. It has been reported that the skeletal muscle expresses ACE2 abundantly [27], which would raise the possibility of direct musculoskeletal damage, as seen in COVID-19, being intrinsically associated with ACE2 expression in the tissue. Moreover, upon entrance into the cells, it is known that SARS-CoV-2 triggers an aggressive inflammatory response that results in damage to multiple structures [28] including skeletal muscle [29]. It has already been established that the entry of the virus through the respiratory airways prompts a strong response to infection, provoking phenomena of dysregulated immunological response, such as the cytokine storm [30]. The storm of cytokines is characterized by lymphopenia and excessive mononuclear cell infiltration in multiple tissues [31,32]. The enormous number of inflammatory markers in the COVID-19 patient’s blood have been linked to increased disease severity and hyper inflammation and an increased neutrophil/lymphocyte ratio, with possible depletion of circulating T-cells. The fact that no changes were observed in articular injuries could be due to the absence of ACE 2 receptors, which are the main target of the virus. This observation could emphasize the possibility that direct musculoskeletal damage, seen in COVID-19, is intrinsically associated with ACE2 expression in the tissue.

In addition, to direct muscle damage by a virus infection, muscle tissue could be negatively affected by other factors related to oxygen supply and cardiovascular impairment. Indeed, the improper activation of neutrophils, observed in patients affected by COVID-19, could represent one of the potential explanations for diffuse microvascular thrombosis and capillary leak syndrome.

The exposure to the viral infection it has been shown that produce a cellular infiltration of neutrophils associated with a decrease in antioxidant defense. Specifically, improper activation of neutrophils, observed in patients affected by COVID-19, could represent one of the potential explanations for diffuse microvascular thrombosis and capillary leak syndrome.

Therefore, an increase in reactive oxygen species (ROS) could damage many circulating cells, such as red blood cells (RBCs). Moreover, it increases their dysfunction in terms of oxygen and carbon dioxide diffusion capacity and their deformability in traveling through the capillaries, thus favoring thrombocytosis, with consequent impairment in gas exchanges [33].

If these conditions involve the skeletal muscle as a metabolizing tissue and are still not known, then further investigations are warranted in this area to understand the possible direct contribution of COVID-19 in causing muscle injury in infected soccer players. Lastly, whether an early resumption of training before RTP could be a potential cause of increased/relapsed injuries should be investigated, taking into account also that the positive period must be considered and managed in a different way from a simple detraining period, as it is caused by a multiorgan infectious pathology, as is COVID-19 [17]. This study has some limitations. Firstly, the lesions were collected from a single database, even if validated by previous research and referenced for accuracy. In this context, there could be injuries that have not been revealed, omitted, or an interpretation different from the true data. Furthermore, this study focused only on the data of injuries in the sports seasons and were analyzed without considering other external factors that may have contributed to an increase in the risk the injury.

On the other hand, a strength of this study regards the fact that our data showed that the competitive season of the Italian Serie A championship, performed in the post-pandemic lockdown restriction period, had a higher incidence of muscle injuries in soccer players compared to the competitive seasons before the SARS-CoV-2 pandemic.

## 5. Conclusions

In conclusion, considering the observational nature of this study, it is not possible to establish a cause–effect relationship between COVID-19 and muscle tissue damage as has been already observed in other tissues (cardiac, pulmonary, nervous, etc.) [10,19]. Hence, future studies investigating the potential biochemical and morphological effects of SARS-CoV-2 on muscle tissue are strongly needed, along with their related specific management.

## Figures and Tables

**Table 1:** Comparisons between seasons 2016–2017, 2017–2018, 2018–2019, and 2020–2021 with respect to the average number of players and number of official matches played by all Serie A teams.

Seasons					
	(2016–2017)	(2017–2018)	(2018–2019)	(2020–2021)	*p*
Average team players	33 ± 2	32 ± 4	33 ± 3	32 ± 2	0.84
Official match played	44 ± 5	44 ± 6	44 ± 5	44 ± 5	0.99

**Table 2:** Comparisons between seasons 2016–2017, 2017–2018, 2018–2019, and 2020–2021 with respect to average articular and muscular number of injuries of all Serie A teams. Values are given as mean ± SD (* *p* < 0.001, statistically different from season 2016-2017, 2017-2018; ^#^ *p* < 0.01 statistical different from season 2018–2019).

	Season (2016–2017)	Season (2017–2018)	Season (2018–2019)	Season (2020–2021)
Articular injury	10.80 ± 4.03	7.35 ± 3.44	10.05 ± 5.34	7.06 ± 3.93
Muscular injury	15.90 ± 7.23	14.45 ± 5.42	19.30 ± 9.38	28.70 ± 10.48 *^,#^

**Table 3:** Comparisons between seasons 2016–2017, 2017–2018, 2018–2019, and 2020–2021 with respect to average articular and muscular number of injuries of all Serie A teams. Data are presented as mean difference and 95% confidence interval of the difference.

Muscular Injury		Mean Difference	*p*	95% CI
Season (2016–2017)	Season (2017–2018)	1.45	1.000	−5.71 to 8.61
Season (2016–2017)	Season (2018–2019)	−3.40	1.000	−10.56 to 3.76
Season (2016–2017)	Season (2020–2021)	−12.80	0.0001	−19.96 to −5.64
Season (2017–2018)	Season (2018–2019)	−4.85	0.420	−12.01 to 2.31
Season (2017–2018)	Season (2020–2021)	−14.25	0.0001	−21.41 to −7.09
Season (2018–2019)	Season (2020–2021)	−9.40	0.0001	−16.56 to −2.24
**Articular Injury**		**Mean Difference**	***p***	**95% CI**
Season (2016–2017)	Season (2017–2018)	3.45	0.072	−0.18 to 7.08
Season (2016–2017)	Season (2018–2019)	0.75	1.000	−2.88 to 4.38
Season (2016–2017)	Season (2020–2021)	3.20	0.117	−0.43 to 6.83
Season (2017–2018)	Season (2018–2019)	−2.70	0.286	−6.33 to 0.93
Season (2017–2018)	Season (2020–2021)	−0.25	1.000	−3.88 to 3.38
Season (2018–2019)	Season (2020–2021)	2.45	0.430	−1.18 to 6.08

**Table 4:** Comparisons between Player Short Positivity (n = 35) and Player Long Positivity (n = 35) using the median-split technique for days of positivity. Values are given as mean ± SD (* *p* = 0.0014).

	PSP	PLP	Mean Difference	95% CI	ES Interpretation	
Muscular injury	1.31 ± 0.47	1.89 ± 0.90 *	0.57	0.23 to 0.91	0.81	Large

## Data Availability

All study data are included in the present manuscript.
